# Immunosuppressant adherence after heart transplantation: a review on detection, prevention, and intervention strategies in a multidisciplinary

**DOI:** 10.3389/fcvm.2025.1558082

**Published:** 2025-03-10

**Authors:** Ziying Fan, Yong Han, Guojun Sun, Zuojun Dong

**Affiliations:** ^1^ Institute of Pharmaceutical Preparations, Department of Pharmacy, Zhejiang University of Technology, Hangzhou, China; ^2^ Department of Pharmacy, Union Hospital, Tongji Medical College, Huazhong University of Science and Technology, Wuhan, China

**Keywords:** heart transplantation, medication adherence, risk factors, intervention, evaluation methods

## Abstract

Heart transplantation is an effective treatment for end-stage heart disease, and postoperative patients' medication adherence is crucial for transplantation outcomes and long-term survival. By reviewing a large amount of related literature, this review summarizes the current status, evaluation methods, influencing factors, and intervention strategies of medication adherence after heart transplantation, emphasizes the important role of multidisciplinary teams in improving medication adherence, and discusses the importance of transplantation multidisciplinary teams and holistic management. By integrating the research results from various fields such as medicine, pharmacy, psychology, and sociology, it provides a more comprehensive theoretical support and practical guidance for improving medication adherence in heart transplant patients.

## 1 Introduction

Heart transplantation (HTx) is the definitive treatment for end-stage heart failure (1, 2). However, lifelong immunosuppression remains a critical component of solid organ transplantation to prevent transplant rejection due to immune responses (3). Furthermore, good medication adherence is a crucial factor influencing disease outcomes (4), as it is closely associated with patients’ quality of life. The term “adherence” has garnered increasing attention in the medical field (5), while also emphasizing the importance of medication-taking behavior. The World Health Organization defines medication adherence as “the process by which a patient actively, cooperatively, and voluntarily follows the advice of a healthcare provider and takes medication as prescribed” (6).

Medication non-adherence (MNA) is one of the most significant and frequently underestimated modifiable factors affecting the prognosis of transplant recipients (7). Poor medication adherence may demonstrate as delayed or failed initiation of the prescribed regimen, suboptimal implementation of therapy, and premature discontinuation of treatment (6). In an international BRIGHT study, non-adherence with medication management regimens was observed in 82.7% of patients on immunosuppressive medications and 76.1% of patients on general medications within one to five years after HTx (8). Non-adherence to prescribed medication regimens has been linked to an increased risk of adverse medical events, higher rates of hospitalization and emergency room visits, and consequently, elevated healthcare costs (9). This emphasizes the importance of addressing long-term non-adherence to medication for improving long-term prognosis and achieving optimal therapeutic outcomes after transplantation (10).

MNA presents a significant challenge in the field of HTx. Despite the importance of this issue, the optimal diagnostic, prophylactic, and therapeutic strategies for MNA remain unclear. This review critically assesses both traditional and novel approaches to the evaluation, prevention, and treatment of immunosuppressive non-adherence following cardiac transplantation from a multidisciplinary and collaborative perspective. The objective of this review is to critically evaluate the existing evidence regarding the diagnosis, risk factors, and treatment of MNA, with a specific emphasis on aspects that may benefit from a multidisciplinary approach.

## 2 Measurement of medication adherence

Based on an assessment of patient adherence, interventions are developed to enhance patients’ medication-taking behavior. Numerous approaches exist for assessing medication adherence, each with its own unique characteristics, advantages, and disadvantages. The World Health Organization proposes to divides these methods into subjective and objective evaluation techniques (11). As illustrated in Table 1. However, a universally accepted standard for evaluating medication adherence has yet to be established (5).

**Table 1:** MNA measures.

Type	Methodologies	Define	Advantages	Disadvantages
Subjectively	Patient diary	Patient diaries document how patients are following their prescribed therapies.	Simple, convenient and economical	Patient misrepresentation and concealment can adversely affect evaluation results.
	Healthcare professional assessment	Patients were asked to estimate their medication-taking behavior, and HCPs assessed their responses and reactions to determine the level of adherence.	Simple, convenient and economical	The outcome depends on the definition of adherence applied.
Objectivity	Pill counting method	The medication taken by the patient is placed in a special bottle and the amount of medication remaining for a specific period of time is calculated to evaluate the patient's adherence.	Easy to implement	Time-consuming, and various uncertainties about medication loss can lead to overestimation of patient adherence ratings.
	Biological tests	Quantitative analysis of concentrations of drugs, drug metabolites, or markers in biological samples such as blood and urine from patients.	Good accuracy	Limitations in application, invasive and expensive testing, and the willingness of patients to cooperate in the long term are also important issues.
	Electronic monitoring methods	Evaluating patient adherence to medication using modern technological means (electronic components installed in the cap of the medicine bottle to automatically record the exact time and number of times the bottle was opened or ingestible sensors embedded in the pills).	Fast and precise	Expensive equipment and limited applications.
	Prescription drug records	A method for estimating medication adherence based on the number of medications dispensed to patients from a pharmacy drug database.	Easy access to records	Reliability of assessment results relies on completeness and accuracy of medication records.

### 2.1 Subjective measurements

To evaluate patients’ medication adherence, researchers may employ a variety of subjective assessment methods, including direct questioning of patients or caregivers, the use of medication diaries, interviews and healthcare professional assessment. Which are primarily in the form of scales (12). These non-directive measures offer advantages such as simplicity, convenience, and cost-effectiveness, thereby enhancing Healthcare professionals (HCPs) understanding of patients’ attitudes toward medication adherence. However, patients often underreport instances of non-adherence to avoid conflict with healthcare providers, which can lead to an overestimation of medication adherence by HCPs. This inherent limitation poses a significant challenge when using subjective assessment methods.

Most reports of medication adherence in heart transplant patients are self-report scales (13). Of note, the Patient-Reported Outcomes Measurement Information System Medication Adherence Scale has also recently been developed and is currently being validated for its applicability in different diverse pediatric and adult transplant patient populations (14). This is a widely used, free self-reported adherence measure.

### 2.2 Objective measurements

#### 2.2.1 Pill counting method

The pill counting method may be conducted at the patient's outpatient clinic follow-up, telephone follow-up, or at the patient's home. However, adherence detected by this method is often underestimated, particularly in patients with chronic conditions who frequently replenish their medications before they are depleted. Furthermore, the threshold value that differentiates adherence from non-adherence in a study is arbitrarily determined (15), which may result in discrepancies in determining patient adherence and comparing medication adherence across studies. Although pill counting has demonstrated greater accuracy than other subjective methods, since the 1990s, Medication Event Monitoring System has been established as the reference standard for validating other adherence measures, superseding pill counting (15).

#### 2.2.2 Biological tests

The monitoring results can be used as a reference basis for clinicians to judge patients’ medication adherence and help them to determine whether the dosage needs to be adjusted. Calcineurin inhibitors and rapamycin-targeted inhibitors are commonly used medications, and their monitoring can help assess adherence. The most commonly used measures include measurement of the index of variability of drug levels, standard deviation (16, 17), coefficient of variation, and calculation of drug concentration to dose ratio (18–20). Other studies have evaluated them using immunosuppression levels or self-report combined with bioassays (21).

#### 2.2.3 Electronic monitoring methods

The electronic monitoring method employs modern technology to track and assess whether patients are adhering to their prescribed medication regimens. This may be accomplished through the use of electronic pill boxes, smart pill bottles, ingestible sensors, and medication-monitoring systems. Wireless observation therapy has been proposed for the diagnosis of MNA in kidney transplants (22). This study demonstrated the possibility of wireless observational therapy as a potential non-invasive monitoring method but also revealed some problems and side effects. This study showed no serious adverse events or acute rejection during a nine-week follow-up of 20 patients. However, eight patients ended treatment early because they experienced problems, including the occurrence of gastrointestinal symptoms, intolerance to the adhesive personal monitor, or insufficient systemic availability. In addition, seven patients reported developing a rash or fever during the first month of adhesive personal monitor use. Some patients also reported anxiety about continuous monitoring.

#### 2.2.4 Prescription drug records

Prescription drug records represent a methodology for estimating medication adherence, based on records of patient-issued medications from a pharmacy drug database. However, there are some limitations to this approach, such as the inability to detect non-adherence at the individual patient level and the problem of electronic health records interoperability between different health systems (23). In addition, some prescriptions obtained from informal systems may be overlooked, while medication discontinuations that are verbally recommended by physicians but not recorded may be excluded from the dataset. Therefore, in order to provide more comprehensive data, it is recommended that patient adherence information be included in the electronic health records. The Institute of Medicine also recommended in its report that patient adherence information should be included in the electronic health records (24).

According to Mellon et al. (4), the best way to assess medication adherence is to use a combination of techniques. Choosing two or more measurement methods can capitalize on the strengths of each method to more accurately determine the level of medication adherence (4). For example, while the accuracy of the Medication Event Monitoring System method (25) is high, compliance may be overestimated when using this method. Therefore, some studies have used alternative methods, such as pill counting, to validate findings and reduce variation (26, 27). Greater sensitivity and specificity of multiple measures compared with a single measure, particularly self-reported, laboratory assay, and clinician-reported measures (28). A 2020 publication stratified measurement tools for transplant recipients, noting that rich and reliable data can be collected through more direct measures such as electronic monitoring and ingestible smart sensors (13). In conclusion, both subjective and objective evaluations have advantages and disadvantages, and should be employed in conjunction to develop a comprehensive adherence assessment methodology that is more suitable for the use of medications in transplant patients.Similarly, it is important to define valid and clinically useful adherence thresholds (29).

## 3 Risk factors for medication non-adherence in HTx

**Figure 1:**
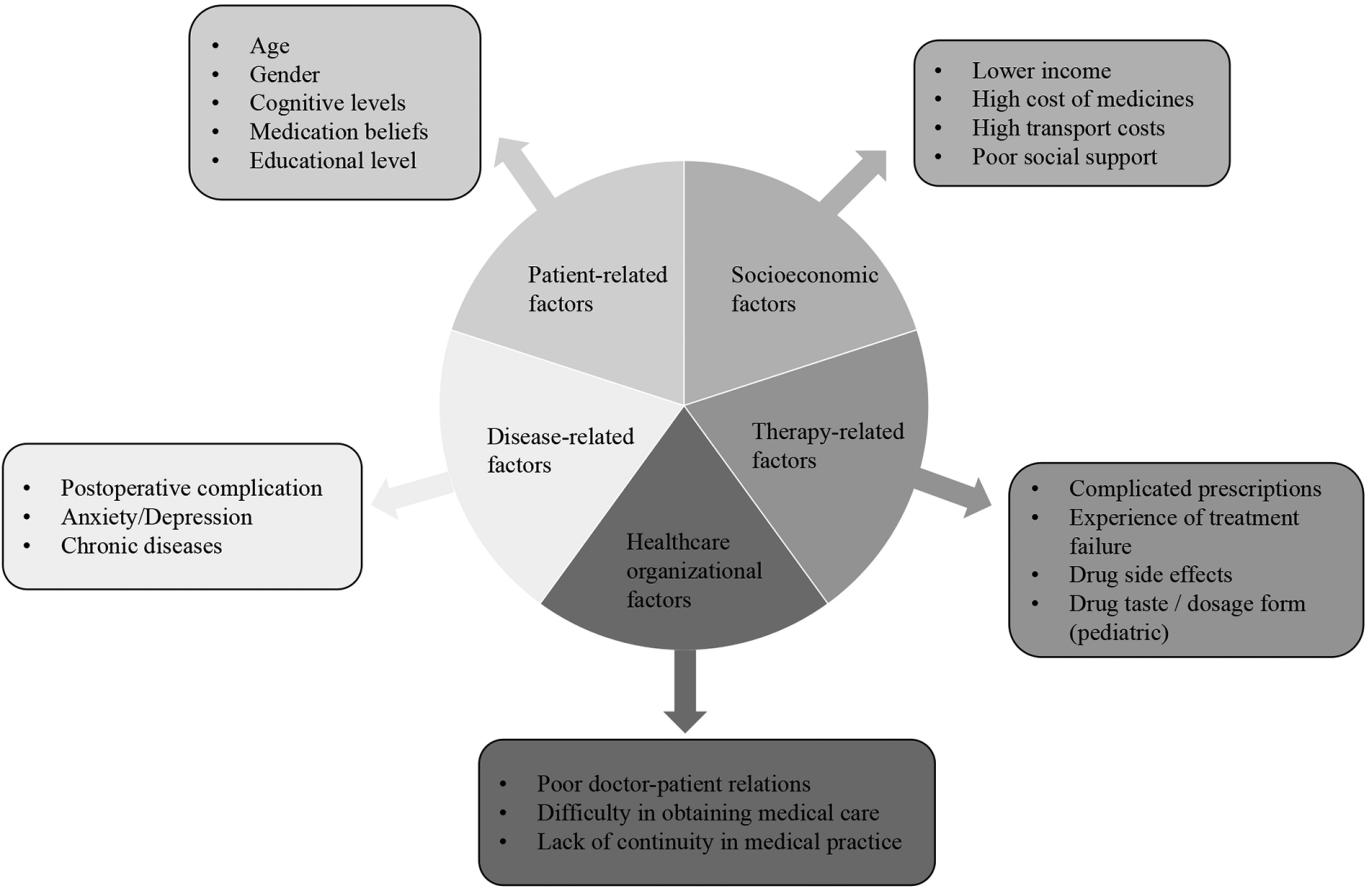
Risk factors for medication non-adherence in heart transplant recipients.

The World Health Organization has developed a conceptual framework that identifies five categories of risk factors for the importance of adherence to chronic disease treatment regimens (11). In reference to this framework, we have compiled a list of risk factors to be examined in heart transplant recipients (Figure 1).

The adherence to medication regimens in organ transplant recipients is influenced by a multitude of factors, including those related to the patient, the disease, the treatment, the socioeconomic context, and the healthcare system (13). Additionally, risk factors for non-adherence can be classified as either modifiable or non-modifiable (30). Patient physical characteristics and disease factors are typically regarded as immutable, whereas treatment complexity and organizational issues can be amenable to modification through intervention. Patient beliefs and psychological factors can also be modified, although this typically necessitates a multi-component approach. Such modifiable risk factors may also be addressed through the implementation of targeted interventions. The risk factors for MNA can coexist and change over time. Therefore, it is essential to monitor these factors on an ongoing basis to address them as soon as they arise.

### 3.1 Patient-related factors

The majority of studies have indicated that advancing age is a protective factor for medication adherence (31–33), with adherence rates typically increasing with age, Patients who are older at the time of surgery are more inclined to adopt beneficial behaviors, such as adherence to diet and regular contact with health care providers (34). However, adolescent patients struggle with medication adherence (35), so transplant centers should pay more attention to potentially modifiable causes of elevated risk of death in young adulthood, and transplant teams should enhance care from adolescence to adulthood (36). The effect of gender on medication adherence is controversial. Previous studies have suggested that male patients are more adherent (37). However, recent research has challenged this notion, suggesting that female patients may in fact serve as a protective factor for medication adherence (38, 39). Higher levels of education are suggestive of improved survival (40). Patients’ level of knowledge and beliefs about medication are modifiable risk factors for adherence, and patients’ level of knowledge and concerns about medication can affect medication adherence (38).

### 3.2 Disease-related factors

It is evident that disease-related factors are strongly associated with postoperative complications, mental and emotional status, and a history of chronic disease (13). It is therefore considered appropriate to categorize HTx recipients as chronically ill, given the necessity for lifelong surveillance of the transplanted organ and the potential for new or existing complications, including hypertension, diabetes, obesity, chronic kidney disease, or cancer. A history of chronic disease has demonstrated that non-adherence is significantly associated with any degree of graft coronary artery disease, particularly in patients with ischemic and idiopathic heart failure, where adherence is significantly higher. Anxiety and depression represent a significant risk factor for poor adherence (41). Studies examining the impact of psychosomatic status on adherence in heart transplant recipients have identified a 41% prevalence of post-transplantation depressive states (42). Additionally, a degree of guilt and self-blame exists among transplant recipients, who often associate the death of the donor with themselves, leading to impaired adherence (32). A review of the literature on kidney transplantation reveals a correlation between cognitive impairment due to depression or cerebrovascular disease and poor adherence in transplant recipients (43).

### 3.3 Therapy-related factors

Complex medication regimens (44), medication side effects, experiences of treatment failure, and issues related to medication flavor and dosage form in transplant recipients during long-term treatment are risk factors for non-adherence (45). The complexity of a medication regimen is influenced by several factors, including the type of formulation, frequency of administration, product-related considerations, and instructions for use of the medication (46). Multimorbid patients are more likely to have non-adherence to medication and complexity of medical therapy (47–49). Unlike adult transplant recipients, pediatric transplant recipients are more affected by factors such as drug flavor and dosage form, thus requiring the development of child-friendly drug formulations to improve adherence and safety (5).

### 3.4 Socioeconomic factors

Lower patient income, high cost of medications, high cost of transportation (distance for patients to obtain medications), and poor social support (e.g., living alone) are all risk factors for medication non-adherence. Social support has been demonstrated to exert a beneficial influence on medication adherence in transplant recipients (50). Transplant recipients who are married and have a stronger relationship with their spouse tend to understand greater social support and demonstrate better adherence (10). A lower Socioeconomic Position is associated with poorer outcomes following HTx. A study was conducted to examine the effects of multimorbidity and Socioeconomic Position on medication adherence following HTx (8). The study revealed that the number of patients treated with free everolimus, lipid modulators, angiotensin-converting enzyme/angiotensin II inhibitors, calcium channel blockers, and loop diuretics, as well as adherence rates, were lower in recipients with a lower Socioeconomic Position, particularly among those who resided alone or had low incomes. This illustrates how both the choice of medication and the accessibility of healthcare resources can have an indirect impact on medication adherence (36).

### 3.5 Healthcare organizational factors

Healthcare organizational factors include issues such as poor physician-patient relationships, difficulty in accessing healthcare, and lack of continuity in medical practice. Interventions by HCPs in the outpatient setting and improving the quality of communication between physicians and patients can improve medication adherence (10). One study has shown that interventions to achieve target adherence significantly improve adherence when patients have others (e.g., clinicians) to help them read health-related materials more frequently (10). Countries such as Belgium and Israel have included the cost of immunosuppressants in public health insurance, thereby reducing barriers to adherence (32, 51). The risk of MNA was 2.3 times higher in patients who were followed up separately in different hospitals compared with those who were always followed up in the same hospital (39). Limited time for healthcare providers to provide information to patients at discharge and the absence of medical staff in the clinic to counsel patients after they forget to take their medications negatively impact medication adherence (52).

## 4 Strategies to improve medication adherence in HTx

In consideration of the modifiable risk factors for heart transplant medication adherence, including patients’ cognitive level and beliefs about taking medication, self-efficacy, and other factors, this article presents a review of eight studies on interventions for HTx medication adherence (Table 2). These studies examine a range of interventions, including educational-behavioral interventions (53, 54), electronic device support (55, 56), and the simplification of the treatment regimen (44, 57). A greater number of studies have been conducted on the risk factors associated with adherence to medication regimens following a heart transplant. However, intervention studies in this area are few and older.

**Table 2:** Studies evaluating interventions to improve immunosuppressant medication adherence in heart transplant recipients.

Authors, Year, Country (Ref.)	Study design	Sample	Intervention	Control^a^	Definition of adherence	Method of adherence assessment	Duration of outcomes assessment	Adherence outcomes	Variables explored	Findings any clinical outcomes^b^
Dew et al., 2004, USA (58)	Quasi experimental study	-*n* = 64HTx, (IG:24 and 20 caregivers; CG:40 and 40 caregivers), one site -6–36 months post-transplant -Age: IG 45.8% <55 years; CG 57.5% <55 years	Variable (depending on patient preferences) web-based intervention with stress & medical regimen management workshops, monitored discussion groups, electronic communication with HTx team	Usual care	5 specific compliance areas: (a) adherence and utilization of all prescribed medications; (b) attended scheduled clinic appointments; (c) Adherence to routine blood work; (d) Frequency of moderately vigorous physical exercise; (e) prescribed diet.	Interview(5 areas to which heart recipients must attend)	4–6 months(IG: 4months; CG: 4–6 months)	Sub-group using the website's medical regimen workshop showed significantly better compliance at follow-up than all other patients in attending clinic appointments, completing blood work and following diet	Website accessibility and user satisfaction; Mental health; QOL;Medical compliance.	Relative to the CG, IG’ depressive and anxiety symptoms, and caregivers’ anxiety and hostility symptoms declined significantly (*p* < 0.05). QOL in social functioning significantly improved
Moro et al., 2008, Spain (54)	RCT	-*n* = 30HTx(IG:15; CG:15), one site -<1 year post-transplant -Age: not stated in the text	Telephone support (telephone contact with a cardiologist from the HTx)	Standard care	Not explicitly stated in the study	Number of calls, contents of inquiries	M = 194 ± 103 days	28 calls; mean call duration 10.2 ± 3.9 min;39.3% medication dosages; 28.6% lifestyle; 25% infectious symptoms; 7% side effects	NA	NA
Doesch et al., 2010, Germany (57)	Quasi experimental study(pre-experimental design)	-*n* = 54HTx, one site -≥6 months post-transplant, and free of acute infection or rejection -Age ≥18 years; M = 46.2 ± 14.1	Conventional twice-daily TAC or CsA to once daily dosing with modified release TAC	NA(Before and after self-comparison)	Any self-reported non-adherence on any item	Self report(VAS and BAASIS)	4 months	Overall non-adherence at baseline for any of the 4 items was 74% vs. 38% after 4 months (*P* = .0001)	NA	NA
Doesch et al., 2013, Germany (44)	Quasi experimental study(pre-experimental design)	-*n* = 76HTx, one site -≥6 months post-transplant, and free from acute infection or rejection for 4 months -Age ≥18 years; M = 46.0 ± 14.4	Conventional twice-daily TAC or CsA to once daily dosing with modified release TAC	NA(Before and after self-comparison)	Any self-reported non-adherence on any item	Self report(VAS and BAASIS)	8 months	Overall non-adherence at baseline for any of the four BAASIS items was 75.0% vs. 40.3% after 8 months (*P* = .0001).	NA	NA
Hollander et al., 2015, USA (53)	Quasi experimental study	-*n* = 7HTx, one site -Varying lengths of time since transplantation -Age 1–18 years	group visits (Two groups were formed: patients less than one year post-transplant and patients greater than one year post-transplant monthly for the patients <1 year post-transplant and every three months for patients >1 year post-transplant.)	NA(Before and after self-comparison)	Not explicitly stated in the study	Patient survey and record of targe t drug levels	6 months (six group visits in the <1-year group and three visits in the >1-year group)	In the <1-year group, all patients reported 100% medication compliance for the entire study period. In the >1-year group, two of three (66%) patients reported 100% medication compliance with one patient reporting a missed-medication rate of <1 month. Serum drug levels were at or near targets for all patients in both groups for the entire study period, suggesting excellent medication compliance.	NA	NA
Shellmer et al., 2016, USA (55)	Quasi experimental study	-*n* = 7 (7 adolescents and 9 caregivers, HTx = 2), one site -Varying lengths of time since transplantation -Age 11–18 years; M = 15	mHealth technology	NA(Before and after self-comparison)	Not explicitly stated in the study	Perception of use, acceptability	6 weeks	90% adolescents endorsed the graphs or logs of missed/late medication dosing as useful and 100% endorsed the remaining features (e.g., medication list, dose time reminders/warnings) as useful.	NA	NA
Dobbels et al., 2017, Switzerland (51)	RCT	-*n* = 205(IG = 103,CG = 102,HTx = 63) ->1 year post-transplant, twice daily TAC -Age ≥18 years; M = 57.6	Theory-based multicomponent staged adapted medication adherence intervention	Usual care	Any self-reported problem (score 1–5) on any of the 4 items	BAASIS, 5-years clinical event-free survival	15 months (3-month run-in period;6-month intervention period; 6-month follow-up period)	At the end of the 6-month intervention period: dose compliance (IG 95.1%; GG 79.1%); time compliance (IG 92%,GG 72%). At the end of the 6-month follow-up period: dose compliance (IG 97.8%; GG 78.6%); time compliance (IG 94.82%, GG 72.8%)	NA	NA
Gomis-Pastor et al., 2021, Spain (56)	RCT	-*n* = 134(IG = 71, CG = 63), one site ->1.5 years post-transplant -Age ≥18 years; M = 57 ± 14	mHealth technology	Standard care	According to the extent to which a patient's actual dosing corresponded to the prescribed dosing regimen (i.e., omissions of single or consecutive doses, delays in medication taking, or self-initiated dose changes, such as a reduction or increase in dosing, are considered non-adherence)	Self-reported(SMAQ,IMTS and BAASIS); immunosuppressive medication blood levels and compliance with visits	Mean follow-up was 1.6 (SD 0.6) years	Significant increase in adherence in adult HTx recipients compared with standard care[composite score 51% vs. 23%,OR = 0.3 (0.1; 0.6), *p* = 0.001]	NA	NA

^a^ In each study using usual care or enhanced usual care as a control, the intervention group received that care as well.

^b^ Variables other than medication adherence in the study.

### 4.1 Educational behavioral interventions

The objective of influencing medication adherence by educating patients about their medications is to increase patient awareness of the importance of immunosuppressive medications. The educational program should include information on the purpose of the medication, dosage, duration of administration, and potential side effects. Furthermore, regular educational interventions (e.g., group discussions, individual counseling) for patients and their families can facilitate the formation of a more robust support network. One study improved patient adherence by reforming the follow-up clinic model, and improved clinic efficiency and peer support (53). Another randomized controlled trial (RCT) found that telephone support improved treatment adherence, adjusted medications, avoided treatment errors, and detected early complications during follow-up visits (54). Although the educational intervention demonstrated efficacy, it is contingent upon the cognitive capacity and information absorption abilities of patients. Consequently, it may occasionally prove inadequate in accounting for individual differences among patients, resulting in some patients remaining unable to fully comprehend it.

### 4.2 Electronic equipment support

To address the problem of patients forgetting to take their medication, electronic reminders, smart pill boxes and other technological tools can be utilized to improve medication adherence. These devices can remind patients to take their medications at the appropriate time and help physicians modify their treatment regimens through data analysis. A study of heart transplant patients showed that the use of a medication management app improved medication adherence and reduced hospitalizations due to inappropriate medication (51). Another study used a mobile app intervention for patients with HTx and showed significant improvements in medication adherence and patient beliefs (56). However, the use of electronic devices can be challenging for older or low-income patients, so developers need to ensure that the devices have user-friendly interfaces and emphasize data privacy issues.

### 4.3 Psychosocial support

Psychological support for patients with poor social support or psychological disorders can help them cope with psychological stress and social isolation. This includes counseling, support groups, and family involvement. To improve psychosocial outcomes for heart transplant recipients and family caregivers, one study used a comprehensive web-based intervention (58). This intervention combined a web-based stress intervention with a medical program management workshop and used monitoring discussion groups and electronic communication with the transplant team to assess mental health. The study found that patients and caregivers in the intervention group had significantly fewer symptoms of depression and anxiety, as well as significant improvements in mental health and quality of life. Additionally, a subgroup that utilized the site's medical protocol seminars demonstrated significantly enhanced adherence to outpatient appointments, blood work completion, and dietary compliance compared to all other patients at the follow-up assessment. Furthermore, the utilisation of group visit counselling can facilitate the provision of psychosocial support for transplant patients, thereby enhancing peer support and subsequently improving patient engagement and medication adherence (53). Despite the effectiveness of psychosocial support, this strategy typically necessitates a considerable investment of resources, and its sustainability is often constrained.

### 4.4 Motivational behavioral interventions

A motivational-behavioral intervention is centered on the patient and aims to enhance their self-management awareness. This is achieved through the use of incentive mechanisms that stimulate intrinsic motivation, improve initiative and adherence to medication, and utilize motivational interviewing techniques to assist patients in identifying and resolving adherence barriers. It is recommended that patients be assisted in establishing beneficial medication habits through the provision of constructive feedback and guidance. Pharmacists and nurses may utilize motivational interviewing techniques to facilitate medication adherence by exploring patients’ personal values and goals through one-on-one interviews (59). It is recommended that a personalized action plan be developed to encourage the patient's active participation in treatment decisions. It is recommended that regular feedback and encouragement be provided in order to enhance the patient's sense of accomplishment. In studies of transplant patients, motivational interventions have demonstrated the potential to enhance self-management skills and adherence (51, 60). Patients tend to demonstrate superior medication adherence when goal-oriented interventions are employed. While motivational-behavioral interventions have been demonstrated to be effective, their implementation is contingent upon the patient's initial level of motivation. Consequently, some patients may experience difficulty deriving sufficient motivation from these interventions, necessitating the integration of alternative strategies.

### 4.5 Simplification of treatment programs

For patients who have undergone a heart transplant, the number of medication types and the frequency of administration tends to be high, particularly in elderly patients with multiple comorbidities. Complex medication regimens have the potential to cause confusion and decreased compliance among patients. Consequently, the simplification of the treatment regimen (optimization of drug dosage and duration of administration, combining medications, and reduction of the number of medications taken per day) represents an additional strategy to enhance adherence. Doesch et al. transitioned from twice-daily calcineurin inhibitor therapy to once-daily modified-release TAc therapy by streamlining the medication regimen (44, 57). The two studies were conducted with a four-month and an eight-month follow-up, respectively, and were evaluated using a visual analog scale and Basel Assessment of Adherence to Immunosuppressive Medications Scale (BAASIS). The modified-release TAc was generally well tolerated by the patients and demonstrated a significant improvement in overall adherence as determined by their own before-and-after comparisons (*p* < 0.0001). During the post-transplant follow-up period, physicians can collaborate with the multidisciplinary team to assess and modify the treatment regimen, periodically review the patient's medication plan to ensure its feasibility and efficacy, and educate the patient on the proper management of the simplified regimen. While medication simplification regimens are effective in improving adherence, they must be evaluated meticulously to prevent any potential compromise in efficacy.

There is limited confidence in the information available on the effectiveness of interventions to improve adherence to immunosuppressive therapy. Because of the small sample sizes of these studies, the variety of interventions, and the different methods used to define and measure medication adherence, it is not possible to assess the relative efficacy of different intervention types. A meta-analysis of low-certainty evidence showed that interventions to improve immunosuppressant adherence had an effect on secondary outcomes representing alternative clinical markers of immunosuppressant non-adherence, including self-reported adherence, drug trough concentration levels, acute graft rejection, graft loss, and death, but it may have reduced hospitalizations (4). There is limited confidence in the information available on the effectiveness of interventions to improve adherence to immunosuppressive therapy. Because of the small sample sizes of these studies, the variety of interventions, and the different methods used to define and measure medication adherence, it is not possible to assess the relative efficacy of different intervention types. A meta-analysis of low-certainty evidence showed that interventions to improve immunosuppressant adherence had an effect on secondary outcomes representing alternative clinical markers of immunosuppressant non-adherence, including self-reported adherence, drug trough concentration levels, acute graft rejection, graft loss, and death, but it may have reduced hospitalizations. Therefore, comprehensive and sustained management strategies that include systemic changes such as optimizing medication supply chain management, multidisciplinary teamwork, improved access to care, healthcare system electronic health records and remote monitoring, financial support, and insurance coordination are needed to improve patient medication adherence.

The importance of a multidisciplinary approach in the management of patients with heart failure has been emphasized by several organizations, including statements from the Heart Failure Society of America and the American College of Clinical Pharmacy, as well as guidelines from the American College of Cardiology Foundation/American Heart Association (61, 62). Physicians, pharmacists, and nurses play a critical role in identifying patients with low adherence and providing interventions (11, 63). Physicians, pharmacists, and nurses play a critical role in identifying patients with low adherence and providing interventions (64), which can lead to disruptions in patient care delivery. Patients are frequently situated at the nexus of these disparate professionals (64). Therefore, collaboration and communication among multidisciplinary teams is essential to provide continuity and permanence of care (65). A study conducted in Sudan showed that the involvement of a multidisciplinary team consisting of cardiologists, clinical pharmacists, medical staff, nurses, dietitians, psychologists, and cardiac rehabilitation specialists during heart transplants significantly improved patient adherence to medication regimens (66). Pharmacists were guided by a designated protocol to administer medication without additional consent or refusal from the physician. The results of this study are consistent with those of two other retrospective studies (67, 68). The roles and responsibilities of each member of the multidisciplinary heart transplant team are listed in the references of this review for reference (Table 3). The comprehensive literature review also found that the role of the pharmacist in the transplant team is often overlooked, but they have an integral role in patient management, the importance of which is discussed below.

**Table 3:** Roles and responsibilities of each member of the multidisciplinary heart transplant team.

Profession	Roles and responsibilities
Physicians (Surgeons, Cardiologist, Hospitalist/Intensivist, Transplantation fellow)	Responsible for overall patient medical decision-making and treatment planning, surgery-related decisions and post-operative recovery, diagnosis and treatment of cardiac conditions, and inpatient care and management.
Nurses (Advanced Practice Nurse, Transplant Nurse Specialist, Cardiac Rehab Nurse, other types of registered nurse, Licensed Practical Nurse)	Provides routine care, patient education and psychological support to assist patients with cardiac rehabilitation; provides advanced care services that may include diagnosis, treatment and care coordination.
Pharmacist	Responsible for medication management and medication therapy programs. Includes medication reorganization, medication therapy assessment and monitoring, patient education and problem solving.
Dietitians	Provides nutritional counseling and diet plans.
Psychologists	Provide psychological assessment and counseling.
Psychiatrist	Provision of mental health assessment and treatment
Social worker	Help patients deal with social and emotional issues and provide social support.
Physical therapist	Primarily responsible for evaluating, diagnosing, and treating patients with motor dysfunction to help them regain or improve their motor abilities. HCPs who provide physical therapy to maintain, enhance, or restore motor and physical function that is impaired or threatened by disease, injury, or disability.
Occupational therapist	To help patients improve their mobility in daily life and at work, so that people with impaired or limited physical or mental functioning can better participate in society.

The American Society for Organ Transplantation and the United Network for Organ Sharing have developed recommendations for having a clinical pharmacist on the heart transplant team (69). Pharmacists play an important role in and support of multidisciplinary heart failure teams (70). In the United States and Canada, pharmacist communities and networks have been established that aim to promote the role of pharmacists in transplantation (13). The role of pharmacists in improving diabetes control, achieving blood pressure goals, and adhering to guideline-recommended therapies has been demonstrated through empirical evidence (66, 71, 72). Pharmacists involved in the care of solid organ transplant patients can help identify potential medication problems, enhance therapeutic monitoring and promote more effective treatment (73, 74). Their activities include medication redispensing, medication therapy assessment and monitoring, patient education and problem solving. Pharmacist involvement is critical to optimizing medication regimens and promoting patient adherence (75). A study of heart and lung transplant candidates demonstrated the clinical relevance of pharmacist interventions to optimize pre-transplant patient care and preparation (76). Solid organ transplant pharmacists working with registered pharmacists to implement interventions can optimize healthcare resource management and demonstrate benefits through cost savings (77). Pharmacists also play an important role in early post-transplant medication management and medication safety (23, 78–81).

## 5 Discussion

By analyzing existing studies, we found that continuity in the management of transplanted patients is an important issue that involves all phases before, during, and after transplantation. The multidisciplinary team needs to ensure that patients receive comprehensive and specialized care throughout the process. Patients transitioning from pediatric to adult healthcare often face adherence issues characterized by instability, vulnerability, poor judgment and decision-making, risk-taking behavior, and strong emotional reactions (82). Other factors associated with outcomes during this period include cognitive abilities, psychological factors, social factors, demographic factors, and systemic issues (83–85). Disruptions in health care utilization are frequently reported during and after transitions in care (86, 87). Therefore, the multidisciplinary team plays a critical role during the transition and in the different phases of a transplant patient's journey. Through a combination of motivational behavioral interventions, holistic management, and continuous monitoring, the multidisciplinary team can be effective in avoiding intentional MNA. After transplantation, lifelong medication administration and rigorous follow-up are required to monitor graft function and prevent complications, which may require the implementation of well-coordinated multidisciplinary care, patient engagement, and self-management.

Comprehensive analyses have shown that single strategies have limitations in interventions, so research is moving toward multifaceted, phased interventions. Although many intervention strategies show some efficacy in the short term, long-term follow-up studies are needed to determine their continued efficacy. To validate the effectiveness and long-term outcomes of these interventions, randomized controlled trials or prospective cohort studies may be conducted. There are differences in the criteria used to measure medication adherence, as well as a lack of guidelines or expert consensus on the definition and standardization of adherence disorders. The lack of guidelines or consensus among experts is a problem. It is recommended that future studies should attempt to establish uniform assessment criteria to improve the comparability and reliability of study results. In addition, studies should focus on integrating multiple measures to improve the comprehensiveness of patient management and the quality of health and standard of living of transplant patients. Enhancement of international collaborations and multicenter research programs aims to elucidate the relationship between chronic disease management and post-transplant multiorgan adaptation in heart transplant patients, and to emphasize the role of multidisciplinary teams in chronic disease management. At the same time, psychological support, family involvement and the utilization of community resources are enhanced to provide comprehensive support.

## 6 Conclusion

In conclusion, an understanding of patient behavior, psychosocial factors, and clinical risks can assist healthcare teams in optimizing care and improving the prognosis of heart transplant recipients. Strategies to enhance adherence to immunosuppressive medications in heart transplant patients should be founded upon the principles of individualized, comprehensive management and provide comprehensive support for patients, HCPs, and society through a multifaceted approach that effectively integrates adherence-related risk factors, minimizes the utilization of healthcare resources, and improves patients’ quality of life. Future research and practice should focus on more in-depth mechanistic explorations and the implementation of an integrated approach, with an in-depth exploration of the interactions and synergistic effects between different interventions to maximize HTx success and patient quality of life, and to achieve optimal adherence outcomes.
